# Assessing the reliability of an online measure of the temporal binding window of audiovisual integration

**DOI:** 10.3758/s13428-025-02791-3

**Published:** 2025-08-18

**Authors:** Leon Flanagan, Nina M. Zumbrunn, Rebecca J. Hirst, David P. McGovern

**Affiliations:** 1 School of Psychology, Dublin City University, Glasnevin Campus, Dublin 9, Ireland; 2 School of Psychology and Institute of Neuroscience, Trinity College Dublin, Dublin 2, Ireland; 3 Open Science Tools (PsychoPy) Lab, School of Psychology, University of Nottingham, Nottingham, UK

**Keywords:** Multisensory temporal processing, Temporal binding window, Sound-induced flash illusion, Web-based assessment, Audiovisual integration

## Abstract

A growing body of evidence suggests that imprecise multisensory temporal processing is associated with a range of adverse clinical outcomes. Specifically, this research has shown that the temporal binding window of audiovisual integration widens with advancing age, whereas individuals with various neurodevelopmental disorders exhibit distinctive profiles of multisensory processing when compared to their neurotypical counterparts. While this line of research has made significant inroads in emphasising the importance of multisensory processing in both typical and atypical cognition, the field would greatly benefit from the validation of web-based tools for assessing multisensory temporal processing, enabling larger sample sizes and the recruitment of typically hard-to-reach populations. Here, we assessed the reliability of a web-based version of the sound-induced flash illusion (SIFI) and examined the level of agreement between the measurements of the temporal binding window that it produces relative to those derived from the SIFI task performed under standard laboratory conditions. Our results demonstrate that both versions of the task elicit a robust SIFI effect and show a similar dependency on the stimulus onset asynchronies between the audiovisual stimuli. Furthermore, our analysis revealed strong correlations between the estimated measurements of both the width and peak amplitude of the temporal binding window derived from the online and lab-based task versions, confirming the suitability of the web-based task for assessing multisensory function. Future studies should extend the evaluation of this online implementation of the SIFI to clinical populations who exhibit impaired multisensory temporal processing, to assess its suitability for clinical research.

**Supplementary Information:**

The online version contains supplementary material available at 10.3758/s13428-025-02791-3.

## Introduction

In order to faithfully and efficiently represent the environments we inhabit, our brains undertake the intricate task of amalgamating and harmonising sensory input derived from the different sensory modalities. The apparent ease with which we execute this function belies the complexity of the underlying computations. One particularly challenging task that the brain is confronted with is knowing which information we should integrate—i.e. when it reflects the same event in the environment—and which information should remain segregated. A potential strategy for solving this task is to limit integration to sensory cues that arrive at the sense organs at the same time. However, this approach requires the brain to exhibit a certain level of flexibility to accommodate disparities arising from differences in physical transmission and sensory processing times inherent in different sensory signals (e.g. King & Palmer, 1985; Raij et al., 2010; Spence & Squire, 2003). The concept of a “temporal binding window” (Dixon & Spitz, 1980)—the time window within which multisensory stimuli are likely to be perceptually fused—has proven useful for understanding these adaptive tolerances. In recent years, many psychophysical methods have been developed to quantify the dimensions of this temporal binding window (e.g. Colonius & Diederich, 2004; Hairston et al., 2005; Navarra et al., 2005; Zampini et al., 2005; Setti et al., 2011; Stevenson et al., 2012; McGovern et al., 2016), and while the optimal width of the window hinges on the prevailing context and circumstances, it tends to be in the range of 100 to 250 ms (Wallace & Stevenson, 2014).

One important application of these methods has been to reveal group-level differences in the width of the temporal binding window (TBW) and, more broadly, in multisensory temporal processing. For example, there is now robust evidence to suggest that the TBW widens as part of the natural ageing process (e.g. Bedard & Barnett-Cowan, 2016; Diederich et al., 2008; McGovern et al., 2014, 2022) and that this widening is particularly pronounced in instances of pathological ageing (Setti et al., 2011; Chan et al., 2015; Hernández et al., 2019). Meanwhile, a parallel strand of literature has highlighted distinctive profiles of multisensory temporal processing observed in individuals with a range of clinical conditions including autism spectrum disorder (de Boer-Schellekens et al., 2013; Donohue et al., 2012; Foss-Feig et al., 2010; Kwakye et al., 2011; Stevenson et al., 2014), schizophrenia (Foucher et al., 2007; Stekelenburg et al., 2013; Stevenson et al., 2017; Williams et al., 2010) and dyslexia (Hahn et al., 2014; Hairston et al., 2005; Harrar et al., 2014) when compared to neurotypical counterparts. These converging lines of inquiry collectively underscore the pivotal role that multisensory processing assumes in facilitating our effective engagement with our surroundings and suggests that behavioural assessments of multisensory integration may offer insights into the understanding of both typical and disrupted cognitive functioning (see Wallace et al., 2020, for a recent review).

While significant strides have been made in revealing population differences in multisensory integration and linking them to clinical outcomes, this line of enquiry would be greatly abetted by the development of a web-based assessment of multisensory temporal processing. Leveraging online recruitment methodologies presents a number of advantages for researchers, most notably the capacity to amass larger and more heterogeneous participant samples (Stewart et al., 2017), helping to address the persistent issue of inadequate statistical power in scientific studies. The development of online tools is also hugely beneficial for conducting clinical investigations, affording researchers the ability to reach and engage with traditionally hard-to-access populations. For example, the implementation of an online measure of the TBW could prove very useful for assessing multisensory temporal processing in individuals with advanced dementia, who may not be able to travel to a research laboratory or might find the prospect of such a visit off-putting. Being able to assess the multisensory function of participants from the comfort of their own home would also significantly expedite clinical investigations of this nature.

While the potential benefits of online testing are clear, they must be tempered against concerns over data quality. This is particularly the case when considering cognitive and perceptual tasks, which traditionally have been conducted within highly controlled laboratory settings (Waskom et al., 2019). The adaptation of perceptual tasks into online formats requires consideration of the variability arising from differences in hardware, visual display settings and operating systems of the individual participants’ personal computers as well as their choice of web browser (e.g. Anwyl-Irvine et al., 2021; Bridges et al., 2020; Semmelmann & Weigelt, 2017); hence, it cannot be assumed that an online version of a perceptual task will faithfully reproduce the results from a lab-based experiment. Notably, of the research conducted to date assessing web-based measures of perceptual function, some have proven to be robust to this variability (e.g. De Leeuw & Motz, 2016; Leadbeater et al., 2023; Semmelmann & Weigelt, 2017), while others have been less successful (e.g. Sasaki & Yamada, 2019; Semmelmann & Weigelt, 2017).

In the current study, we aimed to investigate the consistency and reliability of a web-based measure of the TBW of audiovisual integration. Specifically, participants were required to complete web- and lab-based versions of a numerosity task designed to induce the sound-induced flash illusion (Hirst et al., 2020; Shams et al., 2000). This illusion involves the perception of two flashes when a single flash is accompanied by two beeps, with increasing time differences between the two auditory stimuli (stimulus onset asynchrony) causing the strength of the illusion to gradually diminish, thereby providing a quantifiable measure of the TBW (Ferri et al., 2018; Foss-Feig et al., 2010; Gavin et al., 2022; McGovern et al., 2022). This paradigm has a notable advantage over alternative measures of the TBW in that it relies on a simple and intuitive judgement being made—participants simply have to indicate whether they saw one or two visual flashes—making it well suited for remote testing environments. Moreover, previous work by us (Gavin et al., 2022; McGovern et al., 2014, 2022; Quinn et al., 2023) and others (e.g. Bidelman, 2016; Di Luzio et al., 2021; Ferri et al., 2018; Foss-Feig et al., 2010; Setti et al., 2011; Stevenson et al., 2012) has effectively employed the sound-induced flash illusion to assess the shape of the TBW in both between- and within-subjects experimental designs. We primarily focus on the better-known *fission* variant of the illusion, as it is most commonly used to measure the TBW, and debate is ongoing regarding whether the lesser-known *fusion* variant of the illusion—in which two flashes accompanied by one beep appear as one flash—reflects the same neural processes as sound-induced fission (e.g. Andersen et al., 2004; Hirst et al., 2020; McGovern et al., 2014; Mishra et al., 2008). Nonetheless, we also examine the consistency of the fusion illusion between online and lab-based versions of the task given the increased interest in this phenomenon in recent years.

### Methods

#### Participants

Twenty-two participants (13 female, age range = 18–53 years, mean (SD) age = 25.1 (9.93) years) volunteered to take part in the study and were recruited primarily from the student population of Dublin City University. This sample size is sufficient to detect an intraclass correlation (ICC) of 0.5—classified as the threshold between “acceptable” and “poor” reliability by Koo and Li (2016)—with at least 80% power (Bujang & Baharum, 2017). Criteria for inclusion in the study were as follows: normal or corrected-to-normal vision and hearing; no personal history of epilepsy or photosensitivity; no personal history of migraines. Participants were also required to be between the ages of 18 and 55 years, with the upper age limit chosen on the basis of previous research indicating age-related effects on multisensory integration (e.g. Laurienti et al., 2006; McGovern et al., 2022; Setti et al., 2011). All participants were naive to the purposes of the study and provided their informed consent via an online consent form prior to their participation. Participants received no compensation for their involvement in the research.

All participants included in the study had self-reported normal or corrected-to-normal vision and hearing, and were asked to rate their self-reported visual/auditory function as “poor”, “fair”, “good”, “very good” or “excellent”; 11 participants reported their vision and hearing as “excellent”, with one participant reporting poor vision and another participant indicating poor hearing. All recruitment and experimental procedures were approved by the School of Psychology Research Ethics Committee, Dublin City University (DCU).

#### Design

The study used a within-subject, counterbalanced design to assess the generalisability of the SIFI across lab-based and online settings. Participants were randomly assigned to complete either the lab or online version of the task first, followed by the alternate version approximately 1 week later. For the online session, participants completed the task on their own computers. This design enabled direct comparison of performance across settings while controlling for potential order effects.

#### Apparatus and stimuli

Stimuli were initially created in PsychoPy v2020.2.10 (Peirce et al., 2022), and this version of the task was used in the laboratory setting via the native PsychoPy desktop application. Stimuli were presented on a Dell P2419H monitor at a refresh rate of 60 Hz and a spatial resolution of 1920 × 1080 pixels. For the online version of the experiment, the PsychoPy code was converted to PsychoJS code via the PsychoPy Builder application and pushed to Pavlovia.org so that it could be run online. In both versions of the task, the size and location of visual stimuli were defined in height units in PsychoPy, such that the diameter of the disc stimulus was 10% of the screen height and was presented halfway between the centrally presented fixation cross and the bottom of the screen (approximately 2° in diameter and 5° below fixation on a standard 14-inch laptop screen, respectively, assuming a viewing distance of ~ 57 cm). Visual stimuli consisted of circular discs presented at maximum luminance for 17 ms (a single video frame), while auditory stimuli consisted of brief auditory tones with a frequency of 3.5 kHz presented for 17 ms.

On each trial the visual flash stimulus/stimuli could be accompanied by one, two or no auditory beeps. Thus, there were six different conditions, representing all possible combinations of flashes and beeps; each condition is herein denoted by the number of flashes and beeps (e.g. the 1F2B condition refers to trials where one flash was accompanied by two beeps). In conditions containing two flashes or two beeps, auditory and visual stimuli were separated by one of eight stimulus onset asynchronies (SOAs; − 400 ms, − 200 ms, − 150 ms, − 100 ms, 100 ms, 150 ms, 200 ms, 400 ms), where positive and negative values indicate visual- and auditory-lead trials, respectively. This was the case for both multisensory and unisensory trials in order to keep the number of trials equal across different conditions.

#### Procedure

Participants received a plain-language statement and consent form via email and, upon consenting to take part, were informed as to whether they were assigned to the lab or online version of the task first, the order of which was counterbalanced across participants. Participants received the same instructions irrespective of which order they completed the tasks, with the experimenter providing the instructions either over the phone (online version first) or in person (lab version first). Detailed instructions were also provided on screen prior to the commencement of the study. For the online version of the task, participants were instructed to complete the experiment in a quiet and dark environment using their own laptop computer, sitting 50–60 cm from the screen on a hard-back chair and keeping their eyes fixated on the fixation cross at all times. Participants were also instructed to set the volume on their computer to 70–80% before beginning the experiment. It was not possible to complete the online version of the task on a mobile phone or tablet device. For the lab version of the task, participants completed the task in a quiet, dark room, positioned approximately 57 cm from the screen, while the volume on the computer was set at 70%.

Following the instructions, participants then completed a short auditory task in which they had to say whether they heard one or two auditory beeps at the same SOAs as used in the main experiment. This task consisted of 40 trials (5 trials per each of the positive SOAs) and took less than 5 min to complete. All participants displayed close to perfect performance on this control task, with no differences between the lab and online versions of the task, and very few errors overall. For the main part of the experiment, participants were required to report how many flashes they perceived and were instructed to ignore the auditory beeps, which were irrelevant to the task. Participants indicated their responses with a key press, pressing the “1” key if they perceived one visual flash and the “2” key if they perceived two visual flashes. On each trial, the visual flash stimulus/stimuli could be accompanied by one, two or no auditory beeps. Thus, there were six different conditions, representing all possible combinations of flashes and beeps. All participants completed 20 trials per SOA for these six different conditions, leading to a total of 960 trials (8 SOAs × 6 conditions × 20 repeats) per participant for each version of the task. The main part of the experiment took approximately 45 min. Altogether, each version of the task lasted approximately 1 h, and participants were regularly prompted at set break screens to take self-timed breaks to avoid fatigue.

#### Data analysis

The fission data were analysed in four complementary ways. First, we conducted repeated-measures analyses of variance (ANOVAs) on the raw data with SOA and task setting as factors to assess whether there was a significant difference between the data collected in the lab and online. Where appropriate, the Greenhouse–Geisser correction was applied to adjust the degrees of freedom in order to correct for violations of the sphericity assumption, and adjusted *p*-values are reported. Second, to establish whether the SIFI effects were driven by perceptual sensitivity (*d′*) or by shifts in decision bias (criterion), we carried out a signal detection analysis. Third, Pearson correlation coefficients were calculated to assess the strength of the relationship between the lab- and web-based estimates of the width and amplitude of the TBW, which were derived from a curve-fitting procedure. Fourth, to quantify the level of test–retest reliability between the measures, intraclass correlations were calculated for the lab and online estimates of the TBW to also account for any systematic differences that might exist between data from the two settings (Bujang & Baharum, 2017). In addition, Bayesian versions of the ANOVAs and correlations were run, allowing us to express the strength of evidence for each effect. All statistical analyses were conducted in JASP (JASP Team, 2023).

To evaluate whether SIFI susceptibility was driven by perceptual sensitivity or response bias, we conducted a signal detection theory (SDT) analysis on participants’ responses across SOAs. In this analysis, trials with two flashes and two beeps (2F2B) were treated as signal-present trials, with a response of two flashes classified as a hit and a response of one flash as a miss. Trials with one flash and two beeps (1F2B) were treated as signal-absent (noise) trials, with reports of two flashes classified as false alarms (see also Vanes et al., 2016; Keil, 2020). For each participant and SOA, we calculated *d′* as the difference between the *z*-transformed hit rate and false alarm rate [*d′* = *z*(H) − *z*(FA)]. The criterion was calculated as − 0.5 × [*z*(H) + *z*(FA)], reflecting the participant’s bias toward reporting two flashes regardless of condition. When hit or false alarm rates were equal to 0 or 1, a standard correction was applied by adjusting the rate to (0.5/*N*) or [(*N* − 0.5)/*N*], where *N* is the number of signal or noise trials, respectively (Macmillan & Kaplan, 1985; Stanislaw & Todorov, 1999).

To establish the degree of similarity between the online and lab-based estimates of audiovisual integration, a curve-fitting procedure was employed to establish estimates of the width and peak amplitude of the temporal binding window in both the group-averaged and individual data using GraphPad Prism for macOS. For the group-averaged data, a Gaussian distribution was fitted to trials from the 2F1B condition (i.e. trials known to lead to the fusion effect), with the standard deviation and amplitude left as free parameters to provide estimates of the width and peak amplitude of the temporal binding window, respectively. Given the asymmetric nature of the temporal binding window associated with the fission effect (e.g. McGovern et al., 2022; Stevenson & Wallace, 2013; Stevenson et al., 2012), trials from the 1F2B condition were separated into auditory- and visual-lead trials, with each side of the binding window fitted with a half-normal distribution, and the resulting parameter estimates were averaged.

The individual curve fit analysis was only conducted for the 1F2B (fission) trials, as many of the participants in the current study did not experience the fusion illusion or only experienced it on a small number of trials. For the individual curve fits, each participant’s data from both the online and lab versions of the task were fitted with both the Gaussian and half-normal distributions described above, and the curve fit that yielded the highest average *R*^2^ across the two versions of the task was used to estimate the width and peak of the TBW for that participant. To ensure that the parameter values derived from the curve fits to the individual data provided meaningful estimates of the TBW, curve fits that produced an *R*^2^ of less than 60% were excluded from the analysis (see also Gavin et al., 2022). This led to the removal of two participants from this stage of the analysis. One further participant was excluded from the peak amplitude analysis because the difference score between the lab and online amplitude estimates was greater than three standard deviations from the group mean. We report statistical tests with and without this outlier included; the exclusion of this outlier did not change the statistical significance of any of the reported analyses. Data from all participants in the study were included in the group-averaged analyses and plots.

## Results

### Fission results

**Fig. 1:**
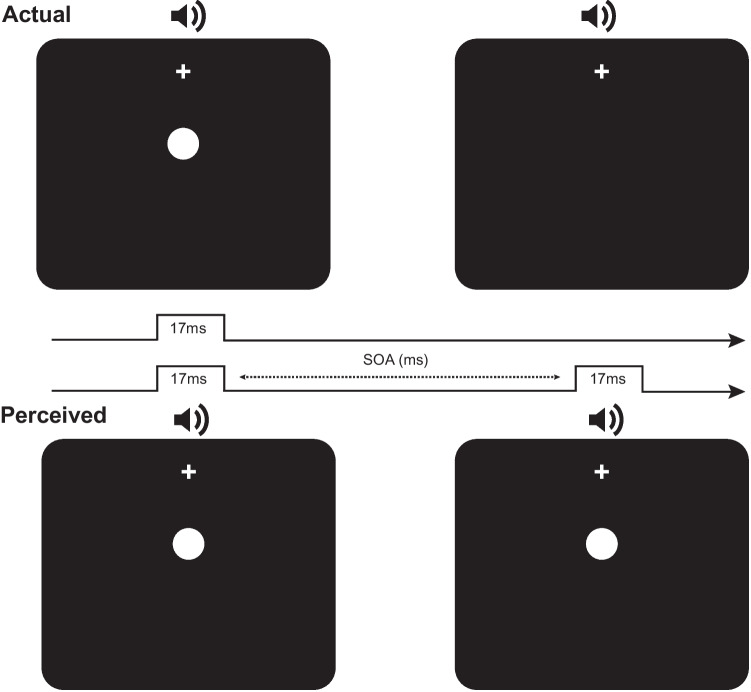
Schematic example of the sound-induced flash illusion. A schematic example of a trial where participants were presented with one visual flash and two auditory beeps (upper part of figure), giving rise to the perception of two flashes (lower part of figure). In this example, there is a positive stimulus onset asynchrony (SOA) such that the visual stimulus is presented with the first auditory beep. Note that the timing of the perceived second flash is illustrative only; it does not imply exact simultaneity with the second beep

**Fig. 2:**
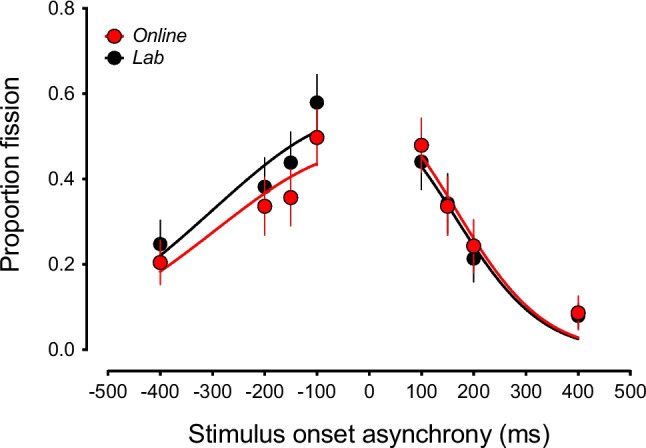
The sound-induced flash illusion as a function of stimulus onset asynchrony (SOA) measured online and in the lab. There is good agreement in the dependency of illusion susceptibility on SOA between the two task versions. *Error bars represent ± 1 SEM*

To assess the reliability of an online measurement of the sound-induced flash illusion, participants in both online and lab-based settings completed a numerosity judgement task where they had to indicate whether they saw one or two flashes, whilst ignoring auditory beeps (see Fig. 1 for schematic). Trials that contained one flash and two beeps (1F2B trials) led participants to experience the fission variant of the SIFI where one visual flash is perceived as two, and Fig. 2 shows that there was good agreement in how the magnitude of the illusion varied according to the SOA of the audiovisual stimuli between the two task versions. In line with this, a repeated-measures ANOVA with SOA and task setting as factors indicated that while there was a significant main effect of SOA (*F*(3.301, 69.328) = 27.395, *p*_adj_ < 0.001, ω^2^ = 0.188, BF_01_ = 1.488 × 10^−21^), there was no significant difference between the versions of the task (*F*(1, 21) = 1.066, *p* = 0.313, ω^2^ = 1.423 × 10^−4^, BF_01_ = 2.94), nor was there a significant interaction between the factors (*F*(3.805, 79.912) = 2.435, *p*_adj_ = 0.057, ω^2^ = 0.005). In keeping with these findings, the results of the half-normal distribution fits to the group-averaged data indicated that both the estimated width (lab, 231 ms; online, 230 ms) and peak amplitude (lab, 0.53; online, 0.51) of the TBW were very similar in the two versions of the task.

**Fig. 3:**
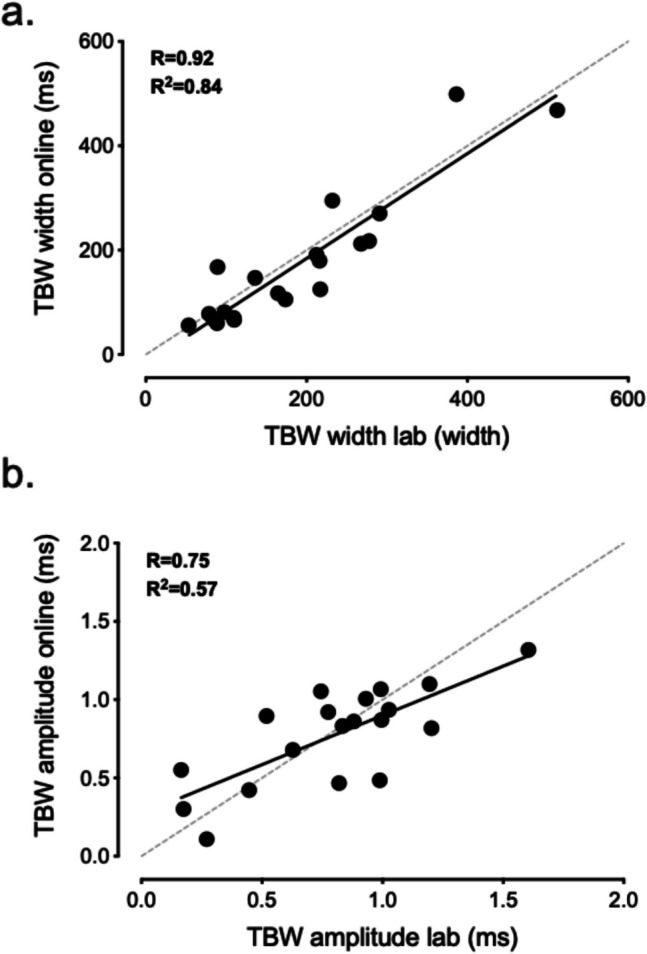
Correlations between lab and online measures of the width (**a**) and amplitude (**b**) of the temporal binding window of audiovisual integration. In both cases, there was a strong relationship in the measurement of these parameter estimates between the lab and online versions of the task. Grey d*otted lines indicate the unity line*

To further quantify the degree of similarity between the two measurements of the TBW derived from the web- and lab-based versions of the task, we fit either half-normal or Gaussian distributions (see *Data analysis* for further details) to each participant’s data to estimate individual measurements of the width and peak of the TBW (see *Supplementary Materials* for individual curve fits). This analysis revealed a strong correlation between the measurements of the width of the TBW derived from the lab and online versions of the task (*r* = 0.92, *p* < 0.0001, BF_10_ = 908,803; see Fig. 3A). Similarly, a strong correlation was found between the web- and lab-based estimates of the peak amplitude of the TBW (*r* = 0.75, *p* = 0.0002, BF_10_ = 165.62; see Fig. 3B), and this correlation remained strong with the inclusion of an outlier that showed a large discrepancy between the amplitude estimates from the lab and online versions of the task (*r* = 0.56, *p* = 0.0096, BF_10_ = 6.32; see *Data analysis* for more details).

To quantify the consistency and reliability of these measurements of the TBW in lab and online settings, we calculated intraclass correlation coefficients (ICCs) for the estimates of the width and amplitude parameter estimates. This analysis indicated that the measurements of the width of the TBW displayed excellent reliability (ICC = 0.91, CI [0.8 0.96]), while the reliability of the amplitude measurements was deemed to be good (ICC = 0.75, CI [0.5, 0.89]; Koo & Li, 2016). Including the outlier described above in this analysis reduced the reliability of this measurement to moderate (ICC = 0.58, CI [0.23, 0.8]).

**Fig. 4:**
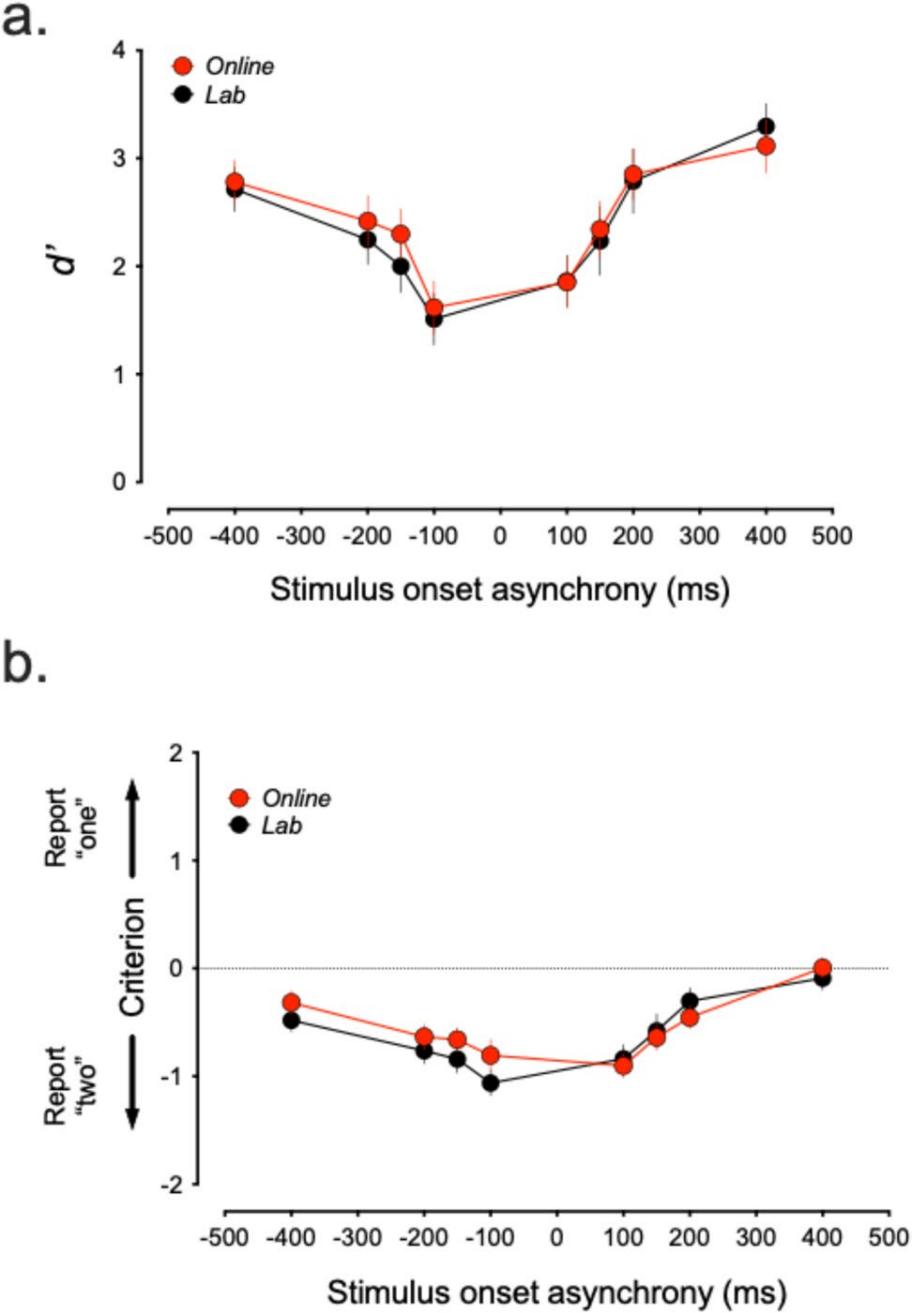
Signal detection analysis of fission responses. Both d′ (**a**) and criterion (**b**) change as a function of SOA, but the changes in d′ are more pronounced. *Error bars represent* ± *1 SEM*

To determine whether the agreement between the lab and online versions of the SIFI was driven primarily by changes in perceptual sensitivity or response bias, we conducted a signal detection analysis on the fission data. Specifically, we calculated *d′* (sensitivity) and criterion (response bias) values for each SOA and setting. This analysis revealed significant effects of SOA for both *d′* (*F*(4.130, 86.729) = 24.772, *p*_adj_ < 0.001, ω^2^ = 0.183) and criterion (*F*(3.251, 68.262) = 23.385, *p*_adj_ < 0.001, ω^2^ = 0.225), indicating that both perceptual sensitivity and decision bias varied systematically with stimulus timing (Fig. 4). However, a complementary Bayesian analysis provided strong evidence in support of the SOA effect on *d′* (BF_10_ = 13.865), with more modest support for the corresponding effect on criterion (BF_10_ = 4.487). By contrast, there was no significant effect of task setting for either *d′* (*F*(1, 21) = 0.371, *p* = 0.549, ω^2^ = 0.0) or criterion (*F*(1, 21) = 1.337, *p* = 0.261, ω^2^ = 0.001), with the Bayesian analysis providing strong support for the null in both cases (*d′* BF_10_ = 2.717 × 10^−20^; criterion BF_10_ = 2.816 × 10^−19^). Together, these findings reinforce the conclusion that the SIFI is predominantly perceptual in nature (see also Watkins et al., 2006; Mishra et al., 2007; Cappe et al., 2010) rather than driven primarily by response bias.

### Fusion results

**Fig. 5:**
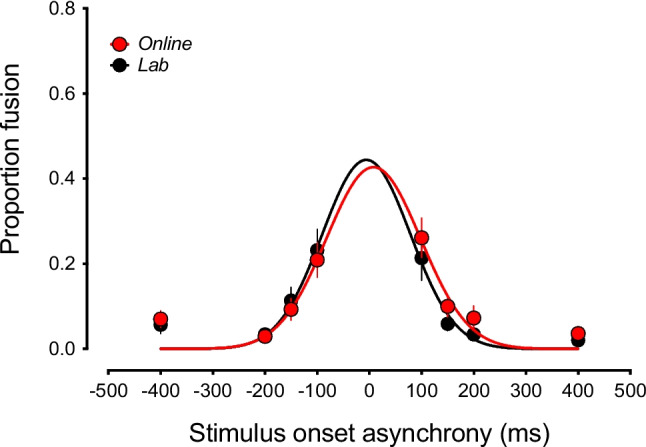
The sound-induced fusion illusion as a function of stimulus onset asynchrony (SOA) measured online and in the lab. There is good agreement in the dependency of illusion susceptibility on SOA between the two task versions. *Error bars represent* ± *1 SEM*

We next assessed the reliability of the fusion variant of the sound-induced flash across lab and online settings. The fusion variant of the illusion is typically experienced by participants on 2F1B trials and tends to be strongest at shorter SOAs leading to the perception of a single visual flash instead of two (Andersen et al., 2004). Figure 5 shows that, like with the fission results, there was very good agreement between the fusion data produced in the lab and online. A repeated-measures ANOVA with SOA and task version as factors indicated that while there was a significant main effect of SOA (*F*(1.8, 37.808) = 16.017, *p*_adj_ < 0.001, ω^2^ = 0.271, BF_01_ = 2.003 × 10^−13^), there was no significant difference between the versions of the task (*F*(1, 21) = 0.533, *p* = 0.474, ω^2^ = 0, BF_01_ = 3.12) nor was there a significant interaction between the factors (*F*(4.441, 93.251) = 1.49, *p*_adj_ = 0.207, ω^2^ = 0.006). In keeping with these findings, the results of the Gaussian distribution fits to the group-averaged data indicated that both the estimated width (lab, 85 ms; online, 90 ms) and peak amplitude (lab, 0.44; online, 0.43) of the window associated with the fusion effect were very similar in the two versions of the task.

## Discussion

A mounting body of evidence suggests that the shape of the TBW of audiovisual integration is influenced by natural and pathological ageing (e.g. Chan et al., 2015; McGovern et al., 2022; Setti et al., 2011), as well as a range of neurodevelopmental disorders (e.g. Foss-Feig et al., 2010; Hairston et al., 2005; Kwakye et al., 2011), supporting the view that the study of multisensory integration could offer valuable insights into both typical and disrupted cognition (Wallace et al., 2020). This line of inquiry could greatly benefit from the validation of web-based tools for assessing multisensory processing that successfully reproduce the outcomes observed in controlled laboratory settings. To this end, we evaluated the consistency and reliability of an online version of the SIFI paradigm developed in PsychoPy and hosted on Pavlovia (Peirce et al., 2022). Participants completed the SIFI task in both a traditional lab-based setting and online, and we assessed the consistency of the estimates of the width and the amplitude of the binding windows associated with the fission and fusion variants of the SIFI. Our results show high levels of agreement in the parameter estimates derived from the group-averaged data for both the fission and fusion illusions in the two settings, while an individual curve fit analysis of the fission data revealed strong correlations in the width and amplitude of the TBW between the lab- and web-based contexts. These results support the use of online measures of the SIFI for assessing the shape of the TBW, potentially paving the way for its utilisation in clinical populations.

Our findings add to a growing body of research aimed at assessing the feasibility of conducting online psychophysical experiments (e.g. De Leeuw & Motz, 2016; Semmelmann & Weigelt, 2017; Bridges et al., 2020; Pronk et al., 2020; Anwyl-Irvine et al., 2021; Leadbetter et al., 2023; Pronk et al., 2023) and complement studies investigating inter-individual variability and the reliability of multisensory integration (Gijbels et al., 2025; Magnotti et al., 2018; Noel et al., 2016). Evaluating the validity of online experimental studies requires careful consideration of several factors that can be broadly categorised into three areas: hardware differences, software differences and environmental differences (Anwyl-Irvine et al., 2021; Semmelmann & Weigelt, 2017). The transition of an experiment from a controlled laboratory setting to an online environment inevitably introduces alterations to both hardware components (e.g. type of computer, monitor, peripherals) and software configurations (e.g. operating system, browser selection, data acquisition software). While we did not attempt to control the hardware settings—aside from restricting participants from using smartphones or tablets to complete the task—we did control some aspects related to the software. For one thing, we chose to run our experiment through Pavlovia, the online extension of PsychoPy (Peirce et al., 2022), which previous research has shown to be a reliable platform for performing online studies (Bridges et al., 2020). Of note, Bridges et al (2020) reported that many system configurations (i.e. combinations of software and operating system) produce a lag in audiovisual stimulus presentation, and that whilst this lag appears consistent within an operating system, variability between system configurations can occur. The former of these issues—i.e. audiovisual stimuli occurring with a consistent lag—may be particularly problematic for studies measuring the TBW if that lag is expected to exceed the width of the TBW. In our study, we did not see evidence of this proving problematic. The issue of device variability is something that might prove more problematic for studies performing between-group comparisons in the TBW, where systematic differences in system configurations between groups could be expected (e.g. if comparing children and adults, and all children use tablets with macOS but all adults use laptops with Windows). Since our study involved within-group as opposed to between-groups comparisons, this was not a limitation for our research question. However, future studies looking to perform between-group comparisons should take this into consideration, either in design planning or in analysis. Furthermore, studies aiming to assess reaction time (RT) measures in online multisensory tasks will need to account for system-specific timing variability, which could otherwise obscure subtle group differences; however, if these technical challenges can be effectively mitigated, RT measures may offer valuable additional insights beyond paradigms based on binary choice outcomes.

We aimed to minimise the environmental differences between the lab and online implementations of the task by providing participants with detailed task instructions—both onscreen and verbally—and participants who completed the online version of the task first received a phone call from the experimenter prior to them beginning the experiment. The inclusion of these instructions appears to have had a positive impact on the online data, and this is illustrated in the remarkable consistency of the average *R*^2^ of the individual curve fits across both settings (online, 0.87; lab, 0.86). Furthermore, the correlation coefficients observed between the standard deviation and amplitude estimates of the TBW were either on par with or exceeded those reported in previous studies assessing the test–retest reliability of various perceptual measures (e.g. Goodbourn et al., 2012; Cappe et al., 2014; Ward et al., 2017). Importantly, the counterbalanced within-subject design enabled us to demonstrate that the reliability of the SIFI estimates holds regardless of whether participants completed the task online or in the lab first. Collectively, these findings suggest that the detailed instructions provided to participants served to uphold the quality of the data collected in the online setting and emphasises the potential value of this step for future online studies.

To examine the perceptual versus decisional contributions to SIFI susceptibility, we conducted a SDT analysis to compute estimates of *d′* and the criterion for each SOA. The analysis showed that both *d′* and criterion varied significantly as a function of SOA. Specifically, *d′* declined systematically with increasing SOA, consistent with reduced perceptual integration of audiovisual signals as the temporal separation between the beeps widened. Criterion values also shifted, becoming more liberal (negative) at shorter SOAs and more conservative at longer SOAs, indicating that participants were more likely to report two flashes when audiovisual events were closely aligned. These findings suggest that while response bias plays a role, the systematic change in *d′* remains the more prominent and interpretable pattern, aligning with prior research linking SIFI strength to neural responses associated with early sensory cortices (e.g. Cappe et al., 2010; Mishra et al., 2007; Watkins et al., 2006). Overall, the SDT results support the conclusion that perceptual processes are the primary driver of SIFI susceptibility, although decision-level factors also modulate responses across SOAs to some degree.

The development of an online measure of the TBW holds significant promise for expediting research into multisensory temporal processing in both hard-to-reach neurotypical groups and clinical populations. As a case in point, a recent application of this method enabled us to provide compelling evidence for a distinct profile of multisensory processing among professional goalkeepers relative to both professional outfield players and age-matched controls (Quinn et al., 2023). This study capitalised on the accessibility of a population of professional football players—a demographic that is difficult to access with conventional lab-based experiments—afforded by the online measure of the SIFI. The advantages of employing web-based assessments of multisensory processing extend even further when applied to clinical populations, offering the potential for frequent longitudinal evaluations with a greatly diminished participant burden and considerably reduced administrative costs. Moreover, online platforms facilitate rapid, concurrent data collection, enabling larger sample sizes while also allowing for more rigorous data privacy regulations which may be desirable for certain populations (Gangé & Franzen, 2023). However, remote testing inevitably forgoes some capabilities of the laboratory—most notably, the ability to acquire millisecond-precision neural signals with electroencephalography (EEG) or magnetoencephalography (MEG) that can provide important insights into the cortical dynamics underlying audiovisual timing and binding (e.g. Foxe & Schroeder, 2005; Molholm et al., 2002; Simon et al., 2017). Furthermore, while the current findings offer promising insights into the feasibility of online evaluations of multisensory temporal processing in a typical young adult population, it should not be assumed that this agreement will also hold for clinical populations who may require more support and could conceivably interact differently with online protocols. Thus, a necessary next step before employing this online tool in clinical settings is to verify that a similar agreement between the lab and online measures of the TBW is observed in specific clinical populations of interest.

A key advantage of the SIFI as a measure of the TBW is that it consists of a simple and intuitive discrimination to be made, namely, whether the participant saw one or two visual flashes. This characteristic not only makes the SIFI well suited for use in remote capacities, but also makes it an appropriate measure for testing clinical populations experiencing cognitive decline where alternative measures of the TBW—such as temporal order judgements and simultaneity judgements—may prove more difficult to grasp. One drawback of the current SIFI implementation is the relatively lengthy administration time (approximately 1 h), which may render it unsuitable for certain clinical populations. To optimise its utility, future research should explore the trade-off between the overall task duration of the SIFI task—by reducing the number of trials per SOA—and data quality. Striking the right balance between data quality and mitigating participant fatigue will help in broadening the appeal and applicability of the online SIFI task.

In conclusion, the current results offer compelling evidence that a web-based assay of the sound-induced flash illusion yields reliable measurements of the TBW of audiovisual integration and provides support for its use in assessing multisensory function in young, neurotypical adults. However, further research is required to confirm whether comparable levels of reliability are observed in older adults and clinical populations before considering the integration of these methods into clinical research studies.

## Supplementary Information

Below is the link to the electronic supplementary material.

Supplementary file1 (DOCX 460 KB)

## Data Availability

The data generated and analysed in the current study can be accessed at https://osf.io/ew7jm/

## Open practices statement

The code used to run the online and lab-based versions of the experimental task and anonymised data are available from https://osf.io/ew7jm/
