# Supplementary material for: Assessing the reliability of an online measure of the temporal binding window of audiovisual integration
Source: Behav Res Methods. 2025 Aug 18;57(9):261. doi: 10.3758/s13428-025-02791-3 (PMC12361292; doi:10.3758/s13428-025-02791-3)
Supplement: Supplementary file 1 — Supplementary file1 (DOCX 460 KB) [file 13428_2025_2791_MOESM1_ESM.docx]

**Figure S1:** Individual curve fits for both in-lab and online task settings. A Gaussian function provided a better fit for some participants, while separate half-normal distributions to the positive and negative SOAs provided a better fit to others.
